# Determination of Plasmid Copy Number Reveals the Total Plasmid DNA Amount Is Greater than the Chromosomal DNA Amount in *Bacillus thuringiensis* YBT-1520

**DOI:** 10.1371/journal.pone.0016025

**Published:** 2011-01-25

**Authors:** Chunying Zhong, Donghai Peng, Weixing Ye, Lujun Chai, Junliang Qi, Ziniu Yu, Lifang Ruan, Ming Sun

**Affiliations:** State Key Laboratory of Agricultural Microbiology, College of Life Science and Technology, Huazhong Agricultural University, Wuhan, China; Loyola University Medical Center, United States of America

## Abstract

*Bacillus thuringiensis* is the most widely used bacterial bio-insecticide, and most insecticidal crystal protein-coding genes are located on plasmids. Most strains of *B. thuringiensis* harbor numerous diverse plasmids, although the plasmid copy numbers (PCNs) of all native plasmids in this host and the corresponding total plasmid DNA amount remains unknown. In this study, we determined the PCNs of 11 plasmids (ranging from 2 kb to 416 kb) in a sequenced *B. thuringiensis* subsp. *kurstaki* strain YBT-1520 using real-time qPCR. PCNs were found to range from 1.38 to 172, and were negatively correlated to plasmid size. The amount of total plasmid DNA (∼8.7 Mbp) was 1.62-fold greater than the amount of chromosomal DNA (∼5.4 Mbp) at the mid-exponential growth stage (OD_600_ = 2.0) of the organism. Furthermore, we selected three plasmids with different sizes and replication mechanisms to determine the PCNs over the entire life cycle. We found that the PCNs dynamically shifted at different stages, reaching their maximum during the mid-exponential growth or stationary phases and remaining stable and close to their minimum after the prespore formation stage. The PCN of pBMB2062, which is the smallest plasmid (2062 bp) and has the highest PCN of those tested, varied in strain YBT-1520, HD-1, and HD-136 (172, 115, and 94, respectively). These findings provide insight into both the total plasmid DNA amount of *B. thuringiensis* and the strong ability of the species to harbor plasmids.

## Introduction

Plasmids are extra-chromosomal DNA elements that can be characterized by their copy number in the host [1], [2]. Plasmids can incorporate and deliver genes by recombination or transposition, thus facilitating genetic exchanges in bacterial populations [1]. In general, plasmids encode a wide variety of valuable functional genes, such as those associated with drug resistance, pathogenicity, and many other important processes. However, plasmids are usually considered nonessential for the survival of bacteria, although they metabolically burden their hosts [2]–[4]. To minimize this metabolic load and coexist stably with their hosts, plasmids must control their replication; accordingly, the plasmid copy number (PCN) of a given plasmid is typically fixed within a given host and under defined cell growth conditions [3], [5].

PCN is defined as the number of copies of a plasmid present per chromosome in a cell [6], [7]. The PCN is a key feature of a plasmid: a PCN can be described either as low (1–10 copies), medium (11–20 copies), or high (>20 copies) [8]. The PCN is mostly controlled by the origin of replication, although host physiology also plays a major role in plasmid replication [5]. However, to date, the full impact of PCN on a host strain is not fully understood, but it is clear that PCN must be considered as a key feature of bacteria and requires further study [4]. The previous studies of PCN have been largely focused on modified small plasmids. There are a few studies on PCN of multiple native plasmids in the same host, but with cruder tools [9].

*Bacillus thuringiensis*, which is the most widely used bio-insecticide, has long been noted for its insecticidal crystal protein (ICP). Most ICP-coding genes are located in the mega-plasmid of the organism [10]–[12]. Typically, *B. thuringiensis* harbors numerous (i.e., 2–12) native plasmids, which vary in size from 2 kb to 600 kb [11], [13], [14]. These plasmids may constitute a substantial amount of the total genetic content of the bacterium, representing 10–20% of the genetic material of the cell [12]. The total plasmid genetic content refers to the sum of the sizes of all plasmids present in an organism. However, plasmids usually have multiple copies in their host. Thus, the total genetic content does not accurately reflect the amount of total plasmid DNA. Accordingly, it seems more appropriate to describe the total plasmid DNA amount as the value of the total (or sum) of plasmid sizes multiplied by the PCN. The resulting total plasmid DNA amount would more accurately reflect the quantitative relationship between plasmid and chromosome in an organism. However, the total plasmid DNA amount present in *B. thuringiensis* remains unknown.

Our group previously isolated *B. thuringiensis* subsp. *kurstaki* strain YBT-1520, which is highly toxic to lepidopteron pests, from China [15]. We have already finished sequencing the whole genome of the strain (unpublished), and found that this strain contains 11 endogenous plasmids with vary sizes and modes of replication and its chromosome consists of 5,401,320 bp. In this study, we determined the PCNs of the 11 plasmids present in strain YBT-1520 at the mid-exponential growing stage (OD_600_ = 2.0) using absolute real-time qPCR. We found that the amount of total plasmid DNA was more than the amount of chromosomal DNA in the strain. We also determined that the PCN varied in different hosts and growth stages.

## Results

### PCNs of 11 plasmids in *B. thuringiensis* strain YBT-1520 at the mid-exponential growing stage

We performed real-time qPCR amplification of the total DNA samples from *B. thuringiensis* strain YBT-1520 harboring 11 plasmids and the plasmid DNA standard of target plasmid simultaneously. Absolute quantification was performed using the standard curves that had been constructed. All PCRs displayed efficiency between 95% and 103% and every reaction was optimized to keep the difference of the amplification efficiencies between the single special gene of the plasmid and chromosomal *aiiA* less than 2%. We also determined the PCN of pBMB67 by selecting three different genes on the plasmid as target fragments. Of note, we reported the sequence of the mega-plasmid pBMB67 in a previous study [16]. We designed three pairs of primers on *rep* (22308–22447), *orf4* (41233–40373), and *orf19* (62239–62365) of pBMB67 to determine the PCNs. The results from this experiment showed that the PCNs of pBMB67 targeted at three different fragments were 21.3, 20.1, and 21.0, respectively, with a relative standard deviation of 3.0%. These findings demonstrated the reliability of our experimental results.

**Figure 1:**
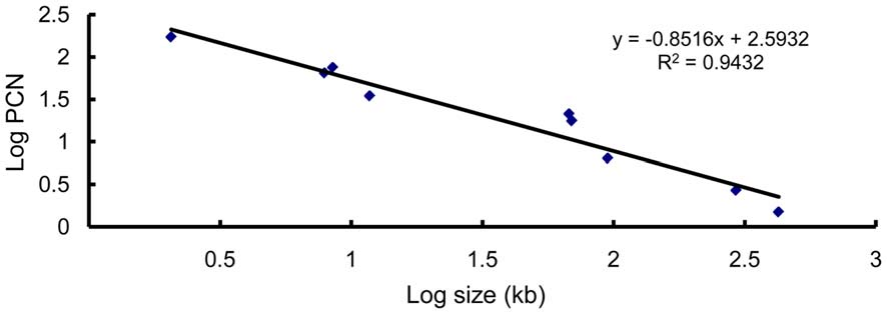
PCN is negatively correlated to molecular mass. The dots from left to right in the graph represent the log values of the size and PCN of pBMB2062, pBMB7921, pBMB8513, pBMB11, pBMB67, pBMB69, pBMB95, pBMB293, and pBMB400, respectively. The PCN and size of each of the nine plasmids in strain YBT-1520 are shown in Table 1.

As shown in Table 1, we calculated the PCNs of the 11 plasmids in strain YBT-1520 at the mid-exponential growing stage (OD_600_ = 2.0) by the absolute quantification method. We found that the PCNs of all 11 native plasmids of strain YBT-1520 ranged from 1.38 to 172 (Table 1). Of note, the largest plasmid pBMB400 had the lowest PCN (1.38) and the smallest plasmid pBMB2062 had the highest PCN (172). The PCN reduced as the molecular weight increased, and thus the PCN negatively correlated to the molecular mass of plasmids (Figure 1). However, the PCNs of some plasmids deviated from this trend. For example, the PCNs of pBMB7635 and pBMB8240 (136, and 139, respectively) were apparently higher than those of pBMB7921 and pBMB8513 (64.7, and 74.7, respectively), although all of these plasmids were similar in size (Table 1). Figure 1 summarizes the relationship between the PCNs of 9 of the 11 plasmids in strain YBT-1520 (except pBMB7635 and pBMB8240) and the plasmid size. We determined that regression analysis of this data presented an R^2^ value of 0.9432.

**Table 1:** PCN of 11 plasmids in strain YBT-1520 determined by real-time qPCR analysis after bacteria grew to the mid-exponential growth stage.

Plasmid	Length (kb)	PCN (M±S.E.)^a^	DNA amount (kb)
pBMB2062	2.062	172±5.67	355
pBMB7635	7.635	136±6.36	1034
pBMB7921	7.921	64.7±1.12	512
pBMB8240	8.240	139±1.76	1148
pBMB8513	8.513	74.7±1.44	636
pBMB11	11.769	34.7±0.69	408
pBMB67	67.730	21.3±1.06	1443
pBMB69	69.416	17.6±0.70	1222
pBMB95	95.150	6.43±0.09	612
pBMB293	293.574	2.72±0.16	799
pBMB400	416.210	1.38±0.14	577
Total	988.21		8746

a Mean ± Standard Error.

In addition, we calculated the plasmid DNA amount by multiplying the plasmid size by the PCN for each of the 11 plasmids from strain YBT-1520. We found that the DNA amounts of the plasmids ranged from 355 kb to 1443 kb (Table 1). Among them, the DNA amounts of pBMB8513, pBMB95, and pBMB400, whose sizes are very different, were quite close to each other (636 kb, 612 kb, and 577 kb, respectively). However, the DNA amounts of pBMB7921 (512 kb) and pBMB8240 (1148 kb), which are similar in size, were quite different.

### PCNs of pBMB2062, pBMB67, and pBMB293 dynamically shifts at different stages during the whole life cycle of strain YBT-1520

**Figure 2:**
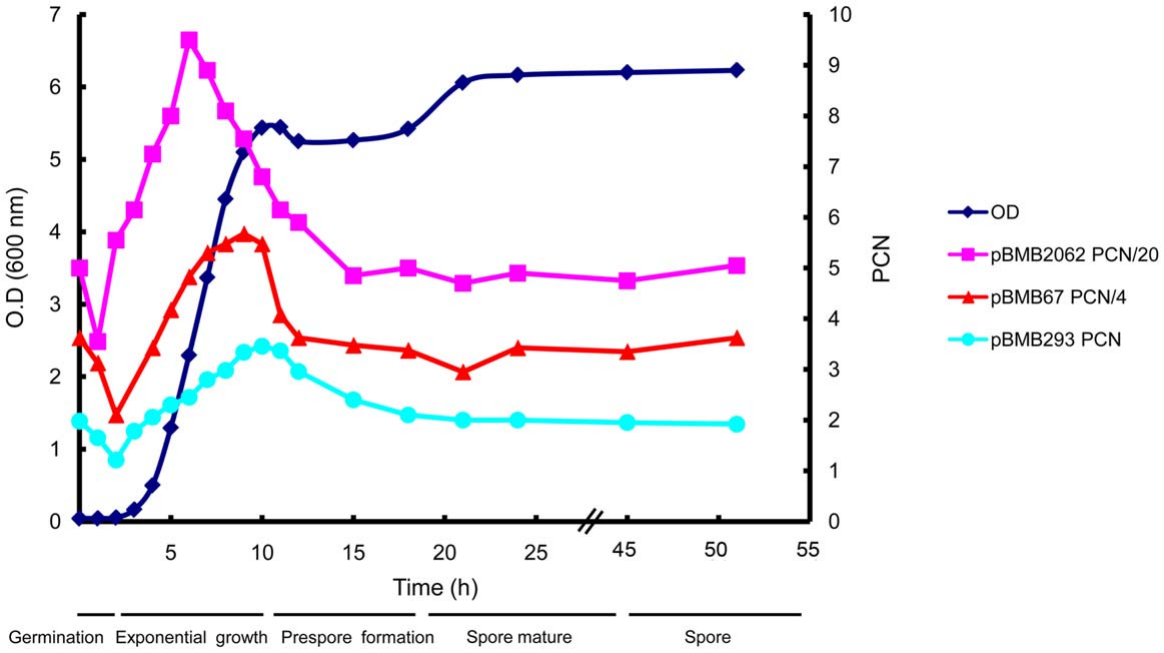
The PCNs of pBMB2062, pBMB67, and pBMB293 vary over the life cycle of *B. thuringiensis* YBT-1520. Spores were inoculated in LB medium to develop into the germination, growth, and spore stages. To show the PCNs of the three plasmids varied widely, we plotted the data for all three plasmid on the same figure: the pink squares represent the PCN of pBMB2062 divided by 20; the red triangles represent the PCN of pBMB67 divided by 4, the aqua circles represent the PCN of pBMB293; and the blue rhombus represent the bacteria's absorbance (OD_600_).

Previous studies reported that the PCNs of some modified small plasmids are different at different stages during the life cycle of the host cell [17], [18]. It has been shown that measurement of the PCN must be performed over the bacteria life cycle without antibiotics in the culture media because the germination, growth, and sporulation of the bacteria are highly affected by the presence of the antibiotics [18], [19]. However, the modified plasmid loss in the absence of antibiotics as a selection pressure could also have a strong effect on PCN measurement. In this study, we selected three native plasmids (pBMB2062, pBMB67, and pBMB293) with different sizes (2 kb, 67 kb, and 293 kb, respectively) and different replication mechanisms [16], [20] to assay the PCNs of these plasmids over the entire life cycle (from spore germination to the spore stage) of strain YBT-1520. We found that the PCNs of all three plasmids dynamically shifted at different growth phases. Specifically, the PCNs ranged from 71 to 190 for pBMB2062, from 8 to 23 for pBMB67, and from 1 to 3 for pBMB293 (Figure 2). The PCNs of all three plasmids were lowest during the germination phase (1–2 h), and then increased rapidly across the logarithmic phase. The PCN of pBMB2062 reached its maximum at the mid-exponential growth stage, while the values of pBMB67 and pBMB293 peaked at the early stationary stage. However, the PCNs of all plasmids decreased rapidly during the late stationary phase and remained stable and close to the minimum after presporulation (18 h) (Figure 2).

### Total plasmid DNA amount is greater than chromosomal DNA amount in strain YBT-1520

To examine the relationship between the total plasmid DNA amount and the chromosomal DNA amount, we calculated the total plasmid DNA amount (Table 1). These data showed that the total DNA amount of the 11 plasmids was equivalent to 8746 kb at the mid-exponential growing stage (OD_600_ = 2.0) (Table 1). To our surprise, the calculated total plasmid DNA amount was 1.62-fold greater than the chromosomal DNA amount (5401 kb).

The data described above showed that the PCNs of all three tested plasmids (pBMB2062, pBMB67, and pBMB293) were close to their respective minimums in the spore stage and remained steady (Figure 2). To further analyze the relationship of total plasmid DNA and chromosomal DNA amounts in the entire life cycle of strain YBT-1520, we calculated the total plasmid DNA amount in the spore stage. The PCNs of the three tested plasmids (pBMB2062, pBMB67, and pBMB293) at this stage were determined to be 100, 14.1, and 1.95, respectively (Table 2), which are close to the corresponding integral numbers (100, 14, and 2, respectively). Accordingly, we deduced that the PCN of pBMB400 was 1 at the spore stage because the PCN at the spore stage was an integral number and was lower than that of the mid-exponential growth stage (1.38). We subsequently compared the PCNs of the four plasmids (pBMB2062, pBMB67, pBMB293, and pBMB400) at the mid-exponential growth stage (OD_600_ = 2.0) to those at the spore stage. These data showed that the ratios (spore stage: mid-exponential stage) of the PCNs of the four plasmids at the different stages were 1.72, 1.51, 1.39, and 1.38, respectively (Table 2). This finding indicates that the ratio decreased slightly with the increase of plasmid size.

**Table 2:** The calculated total plasmid DNA amount when bacteria grew to the spore stage.

Plasmid	PCN of mid-exponential growth stage (M±S.E.)^a^	PCN of spore stage (M±S.E.)^a^	Ratio^b^	Plasmid DNA amount of spore stage (kb)
pBMB2062	172±5.67	100±1.15	1.72	206
pBMB67	21.3±1.06	14.1±0.37	1.51	956
pBMB293	2.72±0.16	1.95±0.02	1.39	572
pBMB400	1.38±0.14	1	1.38	416
pBMB7635	136±6.36	83.0^d^	1.64^c^	634
pBMB7921	64.7±1.12	39.5^d^	1.64^c^	313
pBMB8240	139±1.76	85.1^d^	1.63^c^	701
pBMB8513	74.7±1.44	45.8^d^	1.63^c^	390
pBMB11	34.7±0.69	21.5^d^	1.61^c^	253
pBMB69	17.6±0.70	11.8^d^	1.49^c^	819
pBMB95	6.43±0.09	4.36^d^	1.47^c^	415
Total				5675

a Mean ± Standard Error.

b Ratio of the PCN of mid-exponential growth stage to that of the spore stage.

c Calculation based on a regression (Figure 3).

d Calculation from PCN of mid-exponential growth stage and ratio.

According to the ratios of the PCNs of the four plasmids at the mid-exponential growth (OD_600_ = 2.0) and spore stages (Table 2) and the logged sizes, we fit a linear regression equation (Figure 3). This equation reflected the relationship between plasmid size and the PCN ratio of the two different stages. Therefore, we were able to derive the PCN ratios of the other seven plasmids at the two different stages and their DNA amounts in the spore stage (Table 2). The total plasmid DNA amount of strain YBT-1520 in the spore stage was calculated to be 5675 kb, which was slightly more than the chromosomal DNA amount (5401 kb).

**Figure 3:**
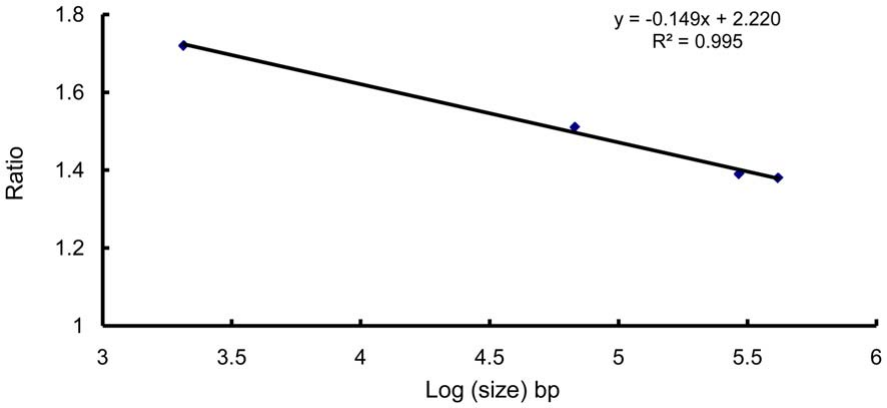
Relationship between the ratio of PCN and plasmid size. The ratio denotes the PCN of pBMB2062, pBMB67, pBMB293, and pBMB400 at the mid-exponential growth stage to that at the spore stage. The ratios of PCNs are also shown in Table 2.

### PCNs of a single plasmid vary in different *B. thuringiensis* hosts

A previous study showed that the PCN of modified plasmids varied from host to host [18]. Furthermore, it was determined that such modified plasmids grew with selection pressure and that antibiotics affected the PCN [18], [19]. Therefore, we measured the PCN of the native plasmid pBMB2062 in three different hosts to further investigate this matter. Of note, pBMB2062 is a widespread cryptic small plasmid. The DNA sequence of this plasmid in strain YBT-1520, HD-1, and HD-136 showed 100% identity. The PCN of pBMB2062 was determined to be the highest (172) among all 11 plasmids in the strain YBT-1520. Therefore, we selected pBMB2062 to monitor the PCN of the same plasmid in different hosts. The PCNs of pBMB2062 in strain YBT-1520, HD-1, and HD-136 were 172, 115, and 94 at the mid-exponential growth stage (OD_600_ = 2.0), respectively. The PCN of pBMB2062 in strain YBT-1520 was almost 1.83-fold of that in strain HD-136. These results indicate that the PCN of a plasmid varies in different hosts, at least for the plasmid included in this study.

## Discussion

Among the 11 plasmids we examined, the PCNs of pBMB7635 and pBMB8240 were very high (Table 1). We previously reported that only the two plasmids of all the 11 plasmids in strain YBT-1520 harbored toxin-antitoxin (TA) systems [21]. TA systems are known to maintain the stability of plasmid in bacteria as addiction systems [22], [23]. Up to now, there is no report about the relationship between the PCN and TA systems. The PCNs of pBMB7635 and pBMB8240 showed high-copy and deviated from the trend that the PCN negatively correlated to the molecular mass of plasmids in this study (Table 1). Whether the TA system will affect the PCNs needs to be validated in the further work.

A previous study indicated that the PCNs of foreign recombinant plasmids were at their minimum during germination and at their maximum during the stationary growth phase during the growth life cycle of *Bacillus cereus* [18]. In the present study, we measured the PCNs of three native plasmids (pBMB2062, pBMB67, and pBMB293) over the entire life cycle of the *B. thuringiensis* strain YBT-1520 (Figure 2). We found that the PCNs were at their minimum during the germination stage for all three native plasmids, and were at their maximum during the stationary growth phase for both pBMB67 and pBMB293. These results are consistent with the previous study [18]. However, the PCN of pBMB2062 reached its maximum at the middle of the logarithmic phase. The PCN of different plasmids reach their maximum at different phase may be due to different plasmid replication regulations or unknown PCN control mechanism on individual plasmid. In addition, there is a dip during germination (1–2 h, actually cell outgrowth within 0.5 h) which is found in previous study also [18]. During this period, the outgrowth cells were adjusting to the changes in the environment; there would be minimal cell growth [24]. The possible reasons for the dip existence might be that the chromosomal DNA replication is earlier than plasmid DNA replication.

The pBMB293 has a pathogenicity island, which contains four ICP genes, *cry1Aa*, *cry1Ia*, *cry2Aa*, and *cry2Ab*. It is interesting that the PCN of pBMB293 reached its maximum at the early stationary growth phase (10 h) and was subsequently reduced to its approximate minimum and stably maintained at 18 h (Figure 2). However, the crystal protein initiate expression at 11 h and reached its maximum at 18 h [25], [26]. Most ICP coding genes are located on low-copy large plasmids [10]. High expression of an ICP should be controlled by its strong promoters, stable mRNA, coexpression of accessory protein, and protein crystallization [26], [27]. In this study, ICPs reach their highest expression level when the PCN is reduced to a level similar to the minimum, indicating that ICP expression may be increased by gene dosage (PCN) but does not have to be proportional. This case was similar as previously reports [26].

In general, bacterial plasmids are considered as “selfish” elements [28]. In other words, their presence is normally a burden for host cells, and thus the host would benefit from elimination of the plasmid intruder [3], [4], [28]. To coexist stably with their hosts, plasmids must control their replication to minimize the metabolic load they exert of their hosts [3], [29]. Plasmids control their PCNs by negative regulatory systems that adjust the rate of replication per plasmid copy in response to fluctuations in the PCN [3]. These regulatory mechanisms have previously been studied in depth [30]–[32]. *B. thuringiensis* strain YBT-1520 contains 11 plasmids; the total plasmid genetic content (988 kb) was determined to be equal to 18.3% of the chromosomal DNA amount (5401 kb), and the total DNA amount of the plasmids (8746 kb) was 1.62-fold more than the total chromosomal DNA amount (5401 kb). It is established that *B. thuringiensis* harbors numerous native plasmids, varying in number from 2 to 12, with sizes from 2 kb to 600 kb [11], [13], [14], and we have shown that the plasmid DNA amount may be more than the total chromosomal DNA amount in this species. These indicate that *B. thuringiensis* strains have a strong ability to harbor plasmids, and that many foreign plasmids could be transferred into *B. thuringiensis* for recombination.

One previous report showed that *Borrelia burgdorferi* B31 contains a linear chromosome of 910,725 bp and at least 17 linear and circular plasmids with a combined size of more than 533,000 bp [33]. The total plasmid genetic content of *B. burgdorferi* B31 (533,000 bp) was found to be 58.5% of the chromosomal DNA amount (910,725 bp). It is reasonable to suggest that the total plasmid DNA amount of *B. burgdorferi* B31 is more than that of its chromosome, also. However, an enormous plasmid DNA amount would inevitably cause great metabolic burden on a host. Thus, one important issue that remains to be resolved is why the total DNA amount of plasmids, which represents an accessory genetic resource, can exceed the chromosomal DNA amount. We have attempted to cure the plasmids in strain YBT-1520 by heat elimination, although we have found that certain plasmids could not be removed (unpublished). In addition, *B. thuringiensis*, especially those strains that are highly toxic to pests, such as YBT-1520, HD-1, H14, etc, contains numerous plasmids [34]–[36]. These information suggest that some plasmids are valuable to permit their hosts to survive better in an adverse environment or to compete better with other microorganisms occupying the same ecological niche.

Bioinformatics analysis of the 11 plasmids in strain YBT-1520 showed that there were many genes present that were likely beneficial to the growth and survival of the organism. For example, pBMB67 may play a role in cell-cell signaling and transcriptional regulation through Rap-Phr cassettes [16]. We speculate that plasmids are more than a burden on their host, and that there may be coevolution and/or a mutualistic relationship present between plasmids and their hosts. This is supported by the ideas that the host spends a lot of energy maintaining the existence of many plasmids and that most plasmids that we examined could not be removed from bacteria.

In conclusion, we demonstrated that the total plasmid DNA amount was greater than the chromosomal DNA amount in the *B. thuringiensis* strain YBT-1520. In addition, we found that the PCN varied at different growth stages and in different hosts. These findings provide us with a new understanding both of the total plasmid DNA amount present and of the strong ability of *B. thuringiensis* to harbor plasmids. Furthermore, our data provide insight into the biological characteristics of plasmids in terms of their variation in both different hosts and different growth stages.

## Materials and Methods

### Bacterial strains, plasmids, and cultural conditions

The bacterial strains and plasmids used in this study are listed in Table 3. The *B. thuringiensis* strain YBT-1520 was isolated in China and the whole-genome was sequenced by a shotgun approach previously (with 2 gaps in chromosome, unpublished data) [15], [37]. All *B. thuringiensis* strains were inoculated from freshly streaked Luria Bertani (LB) agar plates (1% tryptone, 0.5% yeast extract, 0.5% NaCl, and 1.5% agar) cultured overnight into 10 mL of LB media. After incubation at 28°C for 8 h, we transferred 0.5 mL of the culture into 25 mL of LB medium and allowed the bacteria to grow in an incubator shaker (220 rpm) at 28°C until its absorbance (OD_600_) was 2.0. We also used *E. coli* DH5a as the host strain for plasmid amplification. Recombinant *E. coli* was cultured in LB medium (containing 100 µg/mL ampicillin) at 37°C.

**Table 3:** Strains and plasmids used in this study.

Bacteria and plasmids	Characteristic	Source
Bacteria		
YBT-1520	Wild type, *B. thuringiensis* with 11 plasmids, subsp. *kurstaki*	[15]
HD-1	Wild type, *B. thuringiensis* with pBMB2062, subsp. *kurstaki*	[46]
HD-136	Wild type, *B. thuringiensis* with pBMB2062, subsp. *kenyae*	[47]
DH5a	*supE44* △*lacU169 (φ80 lacZ*△*M15) hsd R17 recA1 endA1 gyrA96 thi^-1^ relA1*	Amersham Biosciences
Native plasmids		
pBMB2062	2062 bp, RCR replication, from YBT-1520	NC_002108
pBMB7635	7635 bp, RCR replication, TA system, from YBT-1520	
pBMB7921	7921 bp, RCR replication, from YBT-1520	
pBMB8240	8240 bp, unknown replication mechanism, TA system, from YBT-1520	
pBMB8513	8513 bp, RCR replication, from YBT-1520	
pBMB11	11.77 kb, RCR replication, from YBT-1520	
pBMB67	67.73 kb, θ replication, from YBT-1520	[16]
pBMB69	69.42 kb, θ replication, from YBT-1520	
pBMB95	95.15 kb, θ replication, from YBT-1520	
pBMB293	293.57 kb, unknown replication mechanism, from YBT-1520	
pBMB400	416.21 kb, unknown replication mechanism, from YBT-1520	
pBMB2062-HD1	2062 bp, RCR replication, from HD-1	HM991869
pBMB2062-HD136	2062 bp, RCR replication, from HD-136 (4ac)	DQ185143
Recombinant plasmid		
pGEM-T-*aiiA*	pGEM-T-Easy + *aiiA* (a single copy fragment on YBT-1520 chromosome)	This study
pGEM-T-*aiiA*-2062	pGEM-T-*aiiA* + a single copy fragment on pBMB2062	This study
pGEM-T-*aiiA*-7635	pGEM-T-*aiiA* + a single copy fragment on pBMB7635	This study
pGEM-T-*aiiA*-7921	pGEM-T-*aiiA* + a single copy fragment on pBMB7921	This study
pGEM-T-*aiiA*-8240	pGEM-T-*aiiA* + a single copy fragment on pBMB8240	This study
pGEM-T-*aiiA*-8513	pGEM-T-*aiiA* + a single copy fragment on pBMB8513	This study
pGEM-T-*aiiA*-11	pGEM-T-*aiiA* + a single copy fragment on pBMB11	This study
pGEM-T-*aiiA*-67	pGEM-T-*aiiA* + a single copy fragment on pBMB67	This study
pGEM-T-*aiiA*-69	pGEM-T-*aiiA* + a single copy fragment on pBMB69	This study
pGEM-T-*aiiA*-95	pGEM-T-*aiiA* + a single copy fragment on pBMB95	This study
pGEM-T-*aiiA*-293	pGEM-T-*aiiA* + a single copy fragment on pBMB293	This study
pGEM-T-*aiiA*-400	pGEM-T-*aiiA* + a single copy fragment on pBMB400	This study

### The growth curve during *B. thuringiensis* life cycle

*B. thuringiensis* strain YBT-1520 spores were inoculated into a 500-mL Erlenmeyer flask containing 40 mL of LB medium to a final concentration of 10^6^ spores per mL of LB medium, in order to obtain a growth curve of the organism. The spores were prepared using the previously reported method of Turgeon *et al.* [18]. Before spores were used in experiments, they were incubated at 70°C for 10 min to eliminate remaining vegetative cells. During the strain YBT-1520 life cycle, we collected samples at different times and measured their absorbance at OD_600_ using a Spectrum Lab 23A (LengGuang Tech, Shanghai, China). We also observed sporulation synchronously using an optical microscope. The growth curve includes a plot of absorbance (OD_600_) versus time in culture.

### *B. thuringiensis* total DNA isolation and purification

Total DNA of *B. thuringiensis* vegetative cells was extracted according to the method of Kalman *et al.* [38]. Extracting spores DNA is more difficult than from vegetative, to ensure spores are disrupted, the pellet of spores was resuspended with solution I (25% sucrose, 25 mM Tris-HCl (pH 8.0), 25 mM EDTA), and then ground in liquid nitrogen in a prechilled mortar for 15 min with the constant addition of liquid nitrogen as described by Yoshikawa *et al.* [39] and Rowland *et al.* [40]. Then, we extracted the total DNA from disrupted spores according to the method of extracting vegetative cells DNA. Three separate samples were prepared to reduce the bias of the difference of extraction efficiency between the plasmids and chromosomal DNA. The concentration and quality of extracted total DNA was measured using an UV spectrophotometer DU800 (Beckman Coulter, Fullerton, CA, USA).

### Preparation of plasmid DNA standards for absolute real-time qPCR

We analyzed the strain YBT-1520 genome (unpublished data) and selected a single-copy specific gene as the target gene on the chromosome and 11 plasmids. The chromosomal *aiiA* gene encodes N-acyl homoserine lactone-degrading enzyme, which confers resistance in plants to soft rot disease and other diseases [41], [42]. It was previously determined that chromosomal *aiiA* is a single-copy gene harbored on the chromosome of strain YBT-1520 and many other genomes of sequenced strains of the *Bacillus cereus* species (NC 014171, NC005957). We used chromosomal *aiiA* as a reference gene to detect strain YBT-1520 chromosomal DNA. Most of the plasmid primer pairs were targeted at *Rep* genes and the other plasmids that lacked a specific single-copy *Rep* gene were targeted at other single-copy *orfs*. The primers used in this study are listed in Table 4. All these PCR products were shown to be a single-copy in the strain YBT-1520 genome by a BLAST search against the strain YBT-1520 genome local database. The amplified product of the chromosomal *aiiA* gene was cloned into a pGEM-T Easy vector (Promega, Madison, WI, USA) to produce pGEM-T-*aiiA*. Subsequently, the 11 single-copy fragment of the target plasmid was cloned into pGEM-T-*aiiA* (utilizing restriction endonuclease site for *Pst*I on pGEM-T Easy) to produce 11 plasmid DNA standards, respectively (Table 3). Accordingly, the plasmid DNA standard can be detected using either chromosomal *aiiA* primer pairs or target plasmid primer pairs.

**Table 4:** Primers used in this study.

Primer name	Sequence (5′−3′)	Target gene	Product size (bp)
*aiiA*f	ATACGCCAGGTCATTCTCCA	*aiiA*	138
*aiiA*r	CTGGATCAAATCCTGCGAAC		
p2062f	TTGGAGAGACTCTGACGGTGT	*rep*	123
p2062r	CAAGCTTTTAATGCCCCTTT		
p7635f	TAGGCAAGCTAATGCCGTTC	*orf4*	133
p7635r	CCGTCCACAACAAACAGTAGC		
p7921f	AGACGCGGGTCAGATAATCA	*rep*	132
p7921r	TCCATTCTCTCGCCTTTTTC		
p8240f	TTATCCCCTCCGTTTTCAGG	*orf3*	120
p8240r	TAGTTAAGGCGGAACCAGGA		
p8513f	TGCTGTACAAGCCGCATT	*orf*5	122
p8513r	TAGCCGCTAAACCTGCTACG		
p11f	ACGCAACTCATGCCTAGTCC	*rep*	147
p11r	CGATTGCAGGGTTCTACGAT		
p67-*rep*f	ATCCGTACACCAGCAAAGGA	*rep* (22308–22447)	140
p67-*rep*r	TGCGCTAAAGCACGTAGAGA		
p67-4f	TTCGACAGTTCCAGATGCAG	*orf4* (41233–41373)	141
p67-4r	GCCCCTCTTTCACGCTTAGT		
p67-19f	AGGAATGGAGCCAAAACGAT	*orf19* (62239–62365)	127
p67-19r	TATTTTCCCTGTCGGTGTCC		
p69f	TCAACAAAGAGCATGGCACTA	*rep*	137
p69r	GATCGGCAGCTTGTCTGAAG		
p95f	AACCGCAATGGATTGGATAG	*rep*	124
p95r	AGAAAGCGCTCTTGGAACTC		
p293f	TAACGACCAACTTGCCTTCC	*cry1Aa*	144
p293r	CTGCTTGGCTCAGCATTGTA		
p400f	TCATCCAAACGCCAAGTACA	*orf21*	140
p400r	GCTTGTCATATTCGGCCTTC		

A 10-fold serial dilution series of the plasmid DNA standard, ranging from 1×10^4^ to 1×10^8^ copies/µL, was used to construct the standard curves for both chromosomal *aiiA* and the single-copy fragment of the target plasmid. The concentration of the plasmid DNA standard was measured using a fluorometer and the corresponding PCN was calculated using the following equation [43]. 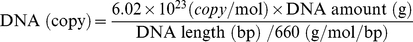



### PCN determination by quantitative real-time PCR

As described previously [6], [44], we calculated the PCN via dividing the target plasmid absolute quantity by the chromosomal absolute quantity in the template total DNA. The IQ5 instrument (Bio-Rad, Hercules, CA, USA) was used for qPCR amplification and detection. Real-time qPCR reactions were performed in triplicate using 25 µL mixtures. The mixture for one reaction contained 2.5 µL of 10× SYBR® Green PCR Master Mix (Toyobo, Kyoto, Japan), 5 µL of template DNA, and 12.5 pmol of each primer. The PCR reaction was conducted for all amplicons with the following cycling conditions: 2 min at 95°C, followed by 40 cycles of 95°C for 15 s, 60°C for 20 s, and 72°C for 30 s. Upon completion of 40 cycles of PCR amplification, a dissociation step of ramping the temperature from 55°C to 95°C steady for 20 min was performed, while the fluorescence signal was continually monitored for melting curve analysis. We determined the cycle threshold (Ct) values after automatic adjustment of the baseline and manual adjustment of the threshold using IQ5 software.

The plasmid DNA standard curve was established according to the method of Lee *et al.* [45]. Briefly, the standard curve includes a plot of the Ct values versus the log concentration of the plasmid DNA standard. For any unknown total DNA sample, the absolute quantity of both its plasmid and chromosomal DNA were obtained by interpolating its Ct value against the standard curve. Subsequently, the PCN was calculated by dividing the copy number of the special gene of a plasmid by the copy number of chromosomal *aiiA*.
